# Photon-counting in dual-contrast-enhanced computed tomography: a proof-of-concept quantitative biomechanical assessment of articular cartilage

**DOI:** 10.1038/s41598-024-78237-1

**Published:** 2024-12-02

**Authors:** Petri Paakkari, Satu I. Inkinen, Ali Mohammadi, Miika T. Nieminen, Anisha Joenathan, Mark W. Grinstaff, Juha Töyräs, Janne T. A. Mäkelä, Juuso T. J. Honkanen

**Affiliations:** 1 Department of Technical Physics, University of Eastern Finland, Kuopio, Finland; 2 Diagnostic Imaging Center, Kuopio University Hospital, Kuopio, Finland; 3 HUS Diagnostic Center, Radiology, Helsinki University and Helsinki University Hospital, Helsinki, Finland; 4 Department of Biomedical Engineering, Chemistry and Medicine, University of California, Davis, CA, USA; 5 Research Unit of Health Sciences and Technology, University of Oulu, Oulu, Finland; 6 Department of Diagnostic Radiology, Oulu University Hospital, Oulu, Finland; 7 Departments of Biomedical Engineering, Chemistry and Medicine, Boston University, Boston, MA USA; 8 Science Service Center, Kuopio University Hospital, Kuopio, Finland; 9 School of Electrical Engineering and Computer Science, The University of Queensland, Brisbane, Australia; 10 Radiotherapy Department, Center of Oncology, Kuopio University Hospital, Kuopio, Finland

**Keywords:** Photon-counting detector, Contrast-enhanced computed tomography, Dual-energy computed tomography, Biomechanical indentation, Articular cartilage, Osteoarthritis, Biomedical engineering, Computed tomography, Imaging techniques, Characterization and analytical techniques

## Abstract

This proof-of-concept study explores quantitative imaging of articular cartilage using photon-counting detector computed tomography (PCD-CT) with a dual-contrast agent approach, comparing it to clinical dual-energy CT (DECT). The diffusion of cationic iodinated CA4 + and non-ionic gadolinium-based gadoteridol contrast agents into ex vivo bovine medial tibial plateau cartilage was tracked over 72 h. Continuous maps of the contrast agents’ diffusion were created, and correlations with biomechanical indentation parameters (equilibrium and instantaneous moduli, and relaxation time constants) were examined at 28 specific locations. Cartilage at each location was analyzed as full-thickness to ensure a fair comparison, and calibration-based material decomposition was employed for concentration estimation. Both DECT and PCD-CT exhibit strong correlations between CA4 + content and biomechanical parameters, with PCD-CT showing superior significance, especially at later time points. DECT lacks significant correlations with gadoteridol-related parameters, while PCD-CT identifies noteworthy correlations between gadoteridol diffusion and biomechanical parameters. In summary, the experimental PCD-CT setup demonstrates superior accuracy and sensitivity in concentration estimation, suggesting its potential as a more effective tool for quantitatively assessing articular cartilage condition compared to a conventional clinical DECT scanner.

## Introduction

Photon-counting detectors (PCDs) are an emerging detector technology in the field of computed tomography (CT) imaging. PCDs directly detect and differentiate X-ray photons by energy^1,2^, meaning that only one acquisition with a polychromatic X-ray source is required to gather spectral information. Traditionally, spectral imaging has been achieved using dual-energy CT (DECT) scanners^3^. However, these scanners normally use conventional energy-integrating detectors (EIDs) that cannot differentiate the energy of photons. As a result, two X-ray spectra are needed for spectral imaging^4^. One alternative for conventional EIDs is dual-layer detectors that also use only one X-ray spectrum, but the spectral separation is fixed due to the thickness and conversion mediums of the detector layers^5,6^. In contrast, PCD’s energy bin thresholds can be customized to suit the use case, offering more flexible spectral separation.

Spectral information is necessary for the material decomposition of CT data, enabling the estimation of contrast agent concentrations. This method is widely applied in contrast-enhanced computed tomography (CECT) to improve soft tissue contrast through increased X-ray attenuation. CECT finds specific application in the assessment of articular cartilage and its health, leveraging the already widespread use of CT in musculoskeletal imaging. Osteoarthritis (OA), the most prevalent degenerative joint disease affecting around 300 million people globally^7^, leads to the gradual degeneration of articular cartilage, causing it to wear away slowly and exposing the articulating bones. This process consequently results in pain and joint stiffness. Unfortunately, by the time symptoms manifest, the degeneration of articular cartilage has significantly advanced such that treatment options are limited, as lost cartilage does not regenerate. Early changes related to OA in cartilage are usually asymptomatic, such as microscopic proteoglycan (PG) depletion and collagen network degradation, which lead to tissue softening and compromised function^8^. Early identification of these changes offers the potential to mitigate degradation and, possibly, prevent the onset of OA, particularly in post-trauma scenarios.

Early detection of OA-related changes in articular cartilage is possible by utilizing CECT^9–12^. However, the current clinical contrast agents for cartilage CT imaging are anionic or non-ionic. The diffusion of anionic contrast agents into cartilage depends on the steric hindrance, water content, PG content, and collagen content^13^, while with non-ionic contrast agents, mainly water content and steric hindrance affect the diffusion^14^. Additionally, the signal afforded by anionic contrast agents is inversely related to PG content^15^, whereas the signal obtained with cationic contrast agents directly correlates with the opposite electrical charge of the PGs and is, thus, more sensitive to PG distribution^15–17^. Hence, the use of a mixture of cationic and non-ionic contrast agents holds the potential for the quantitative assessment of two critical health-reflecting properties of the articular cartilage: PG content and porosity. To this end, a novel dual-contrast agent method utilizing cationic and non-ionic contrast agents has been introduced^18–20^.

PCD technology has thus far been successfully utilized in quantifying iodine and gadolinium-based contrast agents in colon^21^, liver^22,23^, and heart^25^ *in vivo*, and in liver^24^ and articular cartilage^26^ *ex vivo*. PCD-CTs have also been utilized successfully in single-contrast agent imaging of larger *in vivo* porcine model^27^ and to assess bone mineral density from *ex vivo* piglet spine phantom^28^. Since PCDs utilize direct conversion of photons to signal compared to indirect conversion used in EIDs, they have also been shown to provide better spatial resolution, contrast, and signal-to-noise ratio, thus, allowing for similar or better image quality with reduced radiation dose in a clinical setting^29–32^. Based on the earlier studies, the first-generation full-body PCD-CTs exhibit better image quality compared to EID-based clinical full-body CTs^24,33,34^. Thus, PCD-CT has the true potential to be a highly effective technique for situations that involve solving spectral data, such as the presented dual-contrast agent method.

This proof-of-study is a continuation of our earlier study^26^, where we employed PCD-CT to assess osteochondral plugs. Specifically, we assess the biomechanical properties of larger *ex vivo* bovine tibial articular cartilage sample using an upgraded experimental PCD-CT setup. Notably, the larger size of the samples poses a heightened vulnerability to beam-hardening artifacts, a challenge that significantly influences the accuracy of our analysis. Imaging the sample as a function of time reveals the diffusion of the contrast agents into cartilage. Stress-relaxation measurements in indentation geometry yield the biomechanical properties of the cartilage. We hypothesize that despite the challenges posed by larger samples, the experimental PCD-CT system provides superior sensitivity and accuracy compared to a clinical DECT scanner for assessment of articular cartilage biomechanical properties.

## Materials and methods

### Sample processing and measurement locations

A dissected medial tibial plateau (*n* = 1) of skeletally mature bovine was acquired from a local abattoir. No sample-related confounders were controlled. Excessive bone and soft tissues surrounding articular cartilage were removed. Bony surfaces were sealed using cyanoacrylate (Loctite, Henkel Norden AB, Dusseldorf, Germany) so that the contrast agent diffusion would occur only through the articular cartilage surface. Thirty evenly spaced measurement locations were chosen from the load-bearing surface of the tibial plateau. Two of the locations were rejected due to failed biomechanical measurements, leaving 28 locations for the spatial mapping of biomechanical properties and contrast agent distribution analysis.

### Contrast agent solutions

A dual-contrast agent bath was prepared with a total volume of 150 mL, approximately 10 times the volume of the cartilage. The mixture consisted of cationic iodinated CA4 + (*q* =  + 4, *M* = 1499.9 g/mol, 6 mg·I/mL), non-ionic gadolinium-based gadoteridol (Prohance, Bracco International B. V., Amsterdam, Netherlands; *q* = 0, *M* = 558.7 g/mol, 20 mg·Gd/mL), and phosphate-buffered saline with protease inhibitors (ethylenediaminetetraacetic acid disodium salt dihydrate [*C* = 1.86 g/L, VWR International, Radnor, PA, USA] and benzamidine hydrochloride hydrate [*C* = 0.78 g/L, Sigma-Aldrich Co., St. Louis, MO, USA]) to prevent degradation of the sample. The sample was immersed in the dual-contrast agent bath for 72 h at 10 °C to mitigate further degeneration of the samples.

**Fig. 1:**
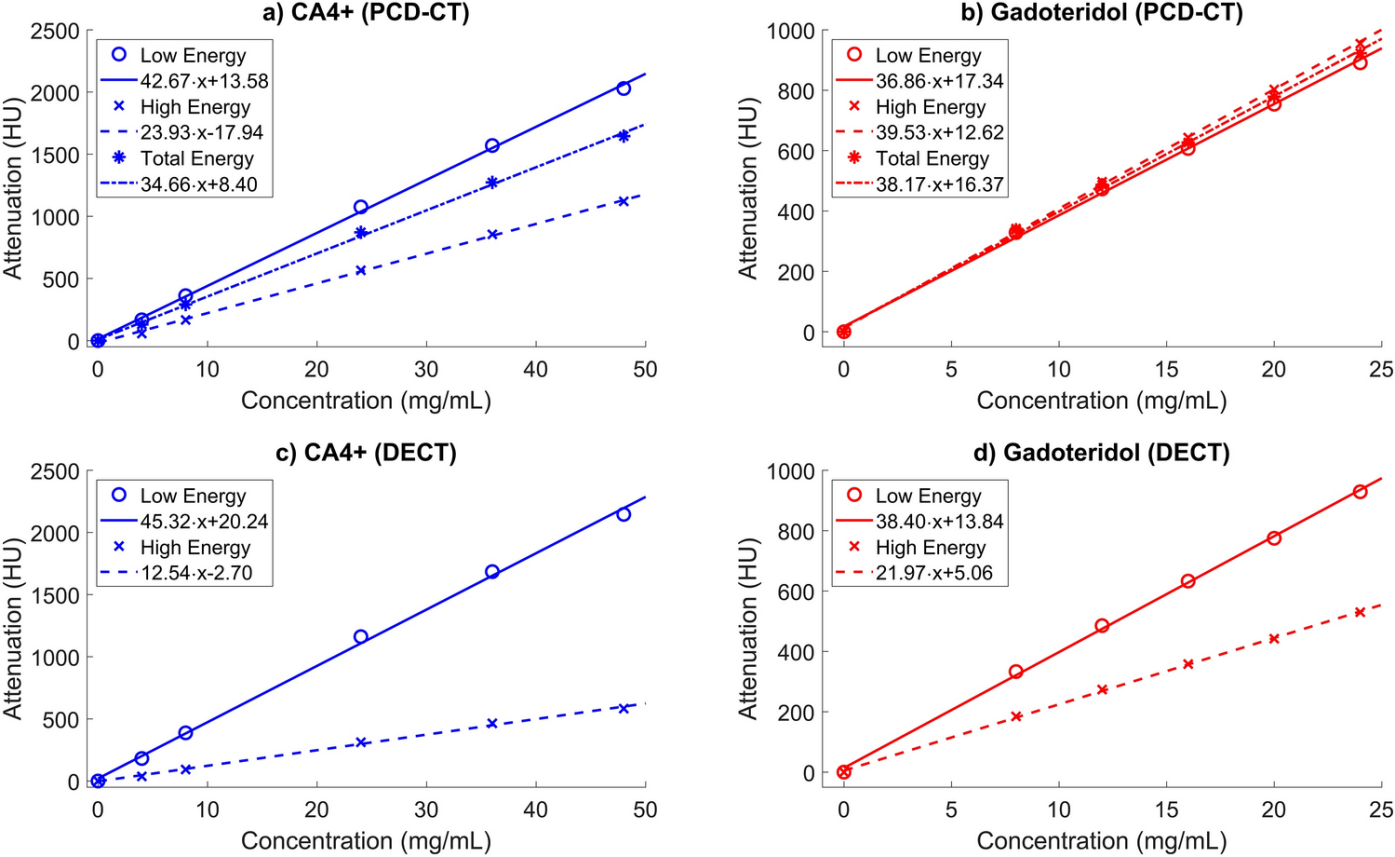
Calibration curves of the CA4 + and gadoteridol for experimental photon-counting detector computed tomography (PCD-CT, **a** and **b**) and clinical dual-energy computed tomography (DECT, **c** and **d**), were used in the concentration estimation. Each calibration solution was imaged with PCD-CT and DECT, and after the Hounsfield unit (HU) conversion, the HUs of the known concentrations were fitted on a linear curve to get a calibration coefficient for the material decomposition. For PCD-CT, three different energy bins (low, high, and total energy) were gathered but only low and total energy calibration curve coefficients were utilized in the concentration estimation.

**Fig. 2:**
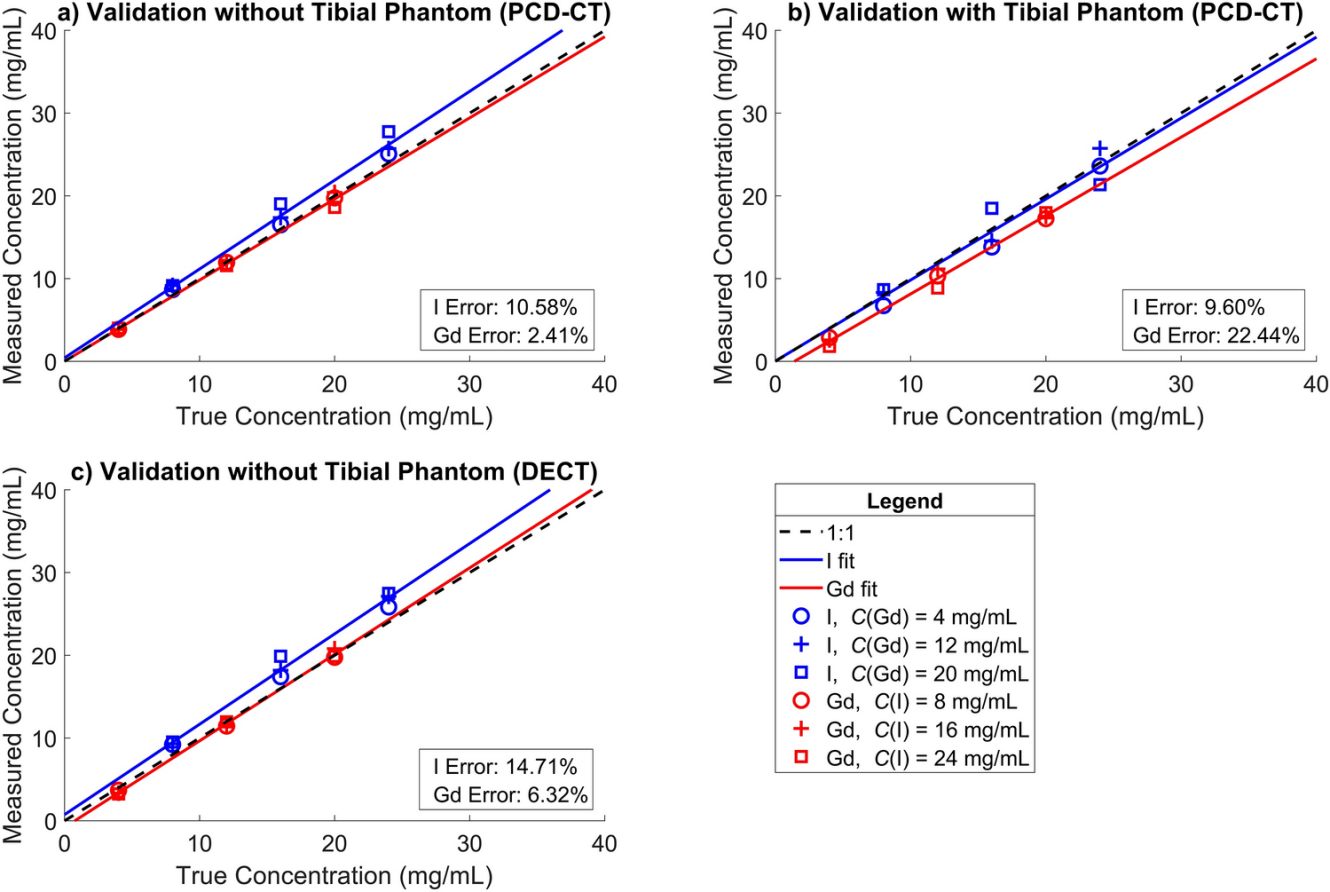
Validation curves for the iodinated CA4 + (I) and gadolinium-based gadoteridol (Gd) estimations for the experimental photon-counting detector computed tomography (PCD-CT, **a** and **b**) and clinical dual-energy computed tomography (DECT, **c**). The PCD-CT validation solutions were imaged with two different setups: with only the mixture tubes imaged (i.e., without tibial phantom), and with tibial phantom next to the tubes (mimicking more realistic conditions, as the contrast agents are estimated inside the sample). The tibial phantom used in the validation measurements was a bovine tibial plateau piece (like the sample used in the contrast agent diffusion measurements). Markers identify different mixtures of contrast agents; one contrast agent concentration was always the same and the other contrast agent concentration was altered (stated on the x-axis). In PCD-CT, low energy and total energy bins were used for the validation and concentration estimations. The reported error is a mean error between the measured concentration and true concentration for that specific contrast agent.

Various concentrations of the contrast agents were prepared for calibration and validation purposes. 2 mL of each calibration solution in different concentrations was administrated in a small vial. The imaged concentrations were 0, 4, 8, 24, 36, and 48 mg·I/mL and 0, 8, 12, 16, 20, and 24 mg·Gd/mL, for CA4 + and gadoteridol, respectively (Fig. 1). 16 mg·I/mL and 4 mg·Gd/mL solutions had to be excluded due to pipetting errors. The validation solutions comprised of known mixtures of CA4 + and gadoteridol, and the volume of each validation solution was 2 mL. The concentration combination of the solutions were 8/4, 16/4, 24/4, 8/12, 16/12, 24/12, 8/20, 16/20, and 24/20 mg·(I/Gd)/mL, respectively, and they were used to validate the calibration data (Fig. 2). The osmolality of the contrast agent mixtures was measured using a freeze point osmometer (Halbmikro-osmometer, GWB, KNAUER Wissenschaftliche Geräte GmbH, Berlin, Germany), and adjusted approximately to 310 mOsm/kg with sodium chloride to mimic the osmolality of saline solution.

### Imaging protocol

The calibration and validation solutions were imaged separately to minimize artifacts like beam hardening in the samples using PCD-CT and DECT scanners. In addition, the PCD-CT setup was used to image the validation solutions with a tibial phantom to introduce artifacts like scattering and beam hardening to the data to simulate the environment where the contrast agents are with the sample. The sample was first imaged with an experimental PCD-CT scanner and then with a clinical DECT scanner at multiple timepoints (0 and 45 min, and 2, 4, 12, 24, 48, and 72 h), but 12- and 48-h timepoints were not included in the DECT data analysis due to imaging failures. The given timepoints do not include the imaging time of the sample (approximately 20 min) since the sample was scanned in air and not in a contrast agent bath. To avoid drying, during PCD-CT imaging, the sample was in a small, sealed plastic bag, and during the DECT scan, the sample was set in an airtight plastic container. Calibration and validation solutions were acquired with both scanners (Figs. 1, 2). Both, EIDs and PCDs, have the potential to suffer from beam hardening, which may impact the accuracy of the results^18,26,35^. Therefore, to assess this issue and study the effect on material decomposition, a tibial phantom was also employed for PCD-CT imaging to create conditions like those in the actual measurements for validation (Fig. 2b). A small water container was present in all the scans to serve as a reference for later analysis.

### PCD-CT setup

The experimental PCD-CT setup consisted of two-bin PCD (XC-Flite FX15, XCounter AB, Stockholm, Sweden), motorized rotator NR360S (Thorlabs Inc., Newton, NJ, USA), and mini-focus X-ray source (IXS1203MF, VJ X-Ray, Bohemia, NY, USA). The X-ray tube had a 50 µm focal spot and the applied tube voltage and current were 120 kVp and 0.25 mA, respectively. The X-ray beam was filtered with 3 mm of aluminum and a removable grid was positioned in front of the PCD to reduce scattering. One PCD-CT scan lasted 12.5 min with a frame rate of two frames per second and a rotation speed of 0.5 degrees per second. In one 360-degree scan, 1440 frames were captured, which equals one frame for every 0.25-degree turn. To increase the signal-to-noise ratio of the projection data, two consecutive frames were averaged, thus, the number of projections in the reconstruction was 720. The bin counter binning thresholds were set to 10 and 60 keV. Only the high energy bin (HE, 60–120 keV), and total energy bin (TE, 10–120 keV) were captured, while the low energy bin (LE, 10–60 keV) was calculated offline. The PCD-CT voxel size in the reconstructed volume was 68.4 × 68.4 × 68.4 μm^3^.

### DECT setup

After each PCD-CT acquisition, the sample was imaged with a clinical DECT scanner (SOMATOM Definition Flash, Siemens, Munich, Germany). The images were acquired using 0.32 × 0.32 mm^2^ in-plane pixel size and the slice thickness was 0.60 mm. The slice thickness was linearly interpolated from 0.60 to 0.32 mm to enable isotropic voxels and direction-independent analysis. DECT images were acquired with two separate scans lasting approximately two minutes together with tube voltages of 70 and 140 kVp. The matrix size was 512 × 512 pixels, the pitch factor was 0.8, the focal spot diameter was 1.20 mm, and the utilized reconstruction kernel was Q40s, which is an edge-enhancing kernel that fits for quantitative analysis. The used tube current and the exposure time for the DECT scanner were 365 mA and 457 ms, and 80 mA and 1000 ms, for the 70 and 140 kVp protocols, respectively.

### PCD-CT data processing

Before image reconstruction, the PCD-CT raw data was preprocessed due to several PCD and imaging-related inhomogeneities and artifacts as follows:

1. Tile-gap interpolation: The PCD consists of two rows of 12 tiles (size per tile: 25.6 × 12.8 mm^2^) which have a one-pixel wide tile-gap. The gaps were filled using linear interpolation from adjacent border pixels.
2. Signal-to-equivalent thickness correction (STC)^36,37^: The X-ray flux intensity to the detector varies with sample thickness and must be corrected. If this is not corrected, the projection image becomes inhomogeneous (non-uniform projection images and ring artifacts in reconstructions). The commonly utilized flat-field correction method is not sufficient for our PCD as the object-free air-image has strong X-ray flux causing, e.g., pile-up effect to the PCD^38^. Therefore, to account for pile-up and possible beam hardening, polymethyl methacrylate (PMMA) was used as the base material in the STC by imaging PMMA plates of various thicknesses (0, 0.5, 1.0, 2.0, 3.9, 6.3, 7.8, 10.5, and 12.5 cm). Then a pixel-wise transformation map was calculated as an exponential attenuation mapping from photon count to PMMA thickness for both energy bins separately. With these maps, the measured counts in the projection data were transformed to an equivalent PMMA thickness.
3. Projection data smoothing: A grid was added in front of the PCD to reduce scattering, however, this caused visible grid lines to the raw projection data. Additionally, our PCD had a few defective pixels. Each projection was post-processed using a Gaussian filter to smooth out the grid lines and defective pixels.

The reconstruction of PCD-CT data was calculated using the Feldkamp, Davis, and Kress method in MATLAB (R2018b, MathWorks, Natick, MA, USA) utilizing ASTRA Toolbox (ver. 1.8.3, Imec-Vision Lab, University of Antwerp, CWI, Amsterdam, Netherlands)^39,40^. Subsequently, the voxel values were transformed from equivalent PMMA thickness to Hounsfield units (HUs) by averaging the area of air and water (roughly 10 × 10 mm^2^) from the image stack and using these averaged PMMA thicknesses values for the HU conversion.

To estimate the contrast agent concentrations from the reconstructed PCD-CT images, a calibration-based material decomposition method was used (Fig. 1a,b)^26^. The concentration estimation was validated using the mixtures with known concentrations by calculating the error between the estimated and the true concentrations (Fig. 2a,b). The concentration estimation was conducted for three energy bin combinations: LE&HE, LE&TE, and HE&TE. Among these, the LE&TE bin solution contained the least error and was chosen to be the deployed energy bin combination for the subsequent analysis. The conversion of HUs to contrast agent concentrations was based on Beer-Lambert law and Bragg’s additive rule, as described earlier^26,41^. Finally, contrast agent partitions were calculated by dividing the measured concentration by the concentration of the original bath^26^.

### DECT data processing

As the DECT data was acquired with a clinical scanner, the data was preprocessed and reconstructed automatically by the software provided by the manufacturer and the reconstructions were already in HUs. The calibration (Fig. 1c,d) and validations (Fig. 2c) were done similarly as to PCD-CT, with the exception that only two energies were used for calibration and no tibial phantom was used for the validation data.

### Imaging data analysis

The biomechanical moduli maps were registered on top of the partition maps using a landmark-based registration to allow comparison of the results. Depthwise partition analysis was conducted perpendicularly to the surface for each location. Depthwise profiles were extracted by averaging a square region-of-interest (ROI) of 21 × 21 pixels (approximately 1.44 × 1.44 mm^2^) for PCD-CT data (Fig. 4). However, due to the DECT’s larger voxel size, the averaged ROI was 5 × 5 pixels (approximately 1.6 × 1.6 mm^2^) to best match the physical ROI size of the PCD-CT. Articulating surface and cartilage-bone interface were identified from the 0-h timepoint PCD-CT TE image stacks using thresholds with fixed HU values: − 500 HU for the surface and 500 HU for the cartilage-bone interface. All profiles were visually checked and then interpolated to have a length of 100 points, and 10% from the beginning and the end of the profiles were removed to minimize the partial volume effect. The same technique was applied to the DECT data, but instead of a depthwise profile, only one bulk value was calculated for each location and contrast agent.

Maximum partition (*P*_max_) and the diffusion time constant *τ* (when 63.2% of the maximum concentration of the contrast agent is reached) were defined for the PCD-CT and DECT data (Fig. 5) by using Eq. (1):

1 $${P}_{diff}\left(t\right)= {P}_{max}\cdot \left(1-{e}^{\frac{t}{-\tau }}\right) ,$$

where *t* is the timepoint.

### Biomechanical measurements

Compressive Young’s equilibrium (*E*_Eq_) and instantaneous (*E*_Inst_) modulus, and relaxation time constants, *α* (fast component of cartilage deformation, reflecting the deformation due to the interstitial fluid) and *β* (slow component of cartilage deformation, reflecting the deformation of the solid matrix) (Eq. 2), were determined with a semi-automated indentation testing protocol. The system comprised a biomechanical tester (Mach-1 v500css, Biomomentum Inc., Laval, Canada) equipped with a high-precision load cell (resolution of 0.005 N, Sensotec Instruments, Barcelona, Spain), an actuator (resolution of 0.1 µm, PM500-1, Newport, Irvine, CA, USA), and a non-porous sphere-headed (*d* = 1.0 mm) indenter. The measurement locations (*N* = 28) were pre-chosen and the stress-relaxation protocol step size was 15% of the cartilage thickness, the strain rate was 100% thickness per second, and the relaxation period was eight minutes. The duration of the relaxation was approved to be long enough that the cartilage is fully relaxed with a preliminary tests. During the indentation, the sample was glued to the bottom of a measuring chamber and the chamber was filled with phosphate-buffered saline. The inclination of the cartilage surface was considered by measuring the surface height at four points around the indentation location and correcting the applied force direction to be perpendicular to the surface. The cartilage thickness at each measurement location was determined using a micro-CT scanner (XT H 225, Nikon Metrology, Leuven, Belgium). The images were acquired with 35 × 35 × 35 µm^3^ isotropic voxel size, 120 kVp tube voltage, and 83 µA tube current without any filters. The thickness was measured perpendicular to the surface.

### Biomechanical analysis

The *E*_Eq_ and *E*_Inst_ were calculated from the stress–strain ratio at mechanical equilibrium and immediately after the ramp phase of the step, respectively, using the solution derived by Hayes et al.^42^. Estimated Poisson’s ratios used in the calculations were 0.2 and 0.5 for the *E*_Eq_ and *E*_Inst_, respectively^43^. Relaxation time constants, *α* and *β*, were determined by fitting the following equation to the time-dependent data:

2 $${f}_{relax}\left(t\right)= a\cdot {e}^{{-\left(t/\alpha \right)}^{\beta }}+d ,$$

where* a* and *d* are fitting constants, and *t* is the time^44^. The time constant *α* is driven by porosity, and in principle, an increase of *α* increases the time to reach relaxation equilibrium. The time constant *β* reflects the relaxation nonlinearity and is believed to be mainly driven by solid content, *i.e.*, strained collagen fibers and PGs; a decrease in *β* shortens the creep, and the relaxation equilibrium is reached at a higher value.

**Fig. 3:**
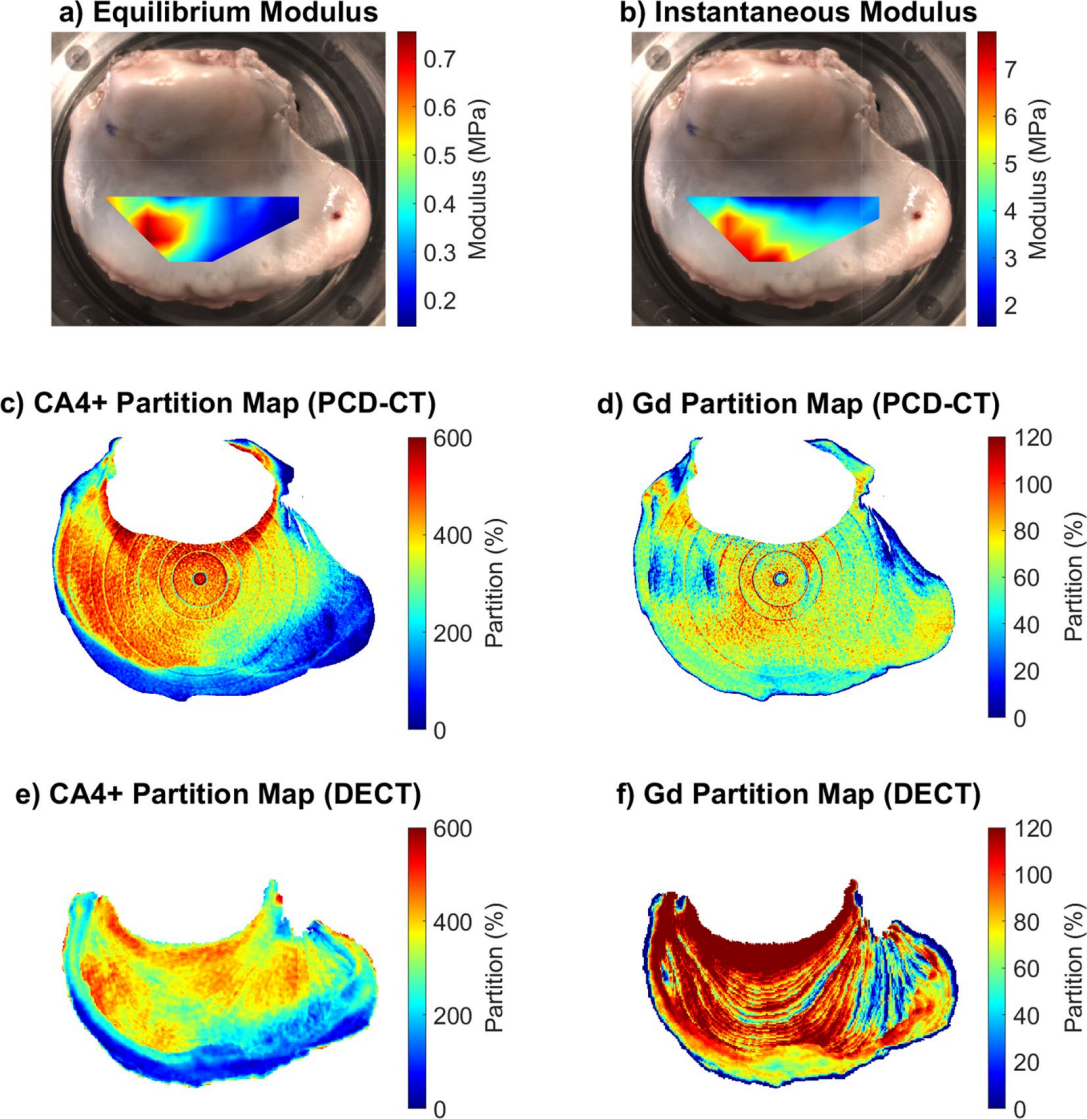
Heatmaps for the biomechanical moduli (**a**,**b**) and the contrast agent partitions of the bovine medial tibial plateau sample (**c**–**f**). The biomechanical moduli heatmaps were calculated by interpolating the data between the measurement locations (*N* = 28). For the partition maps, the cartilage was segmented from the CT data and then partition values were summed through the cartilage in the image stack direction (axial). In the PCD-CT partition maps, some ring artifacts were visible after image processing since the material decomposition strengthens the artifact.

Spatial heatmaps of *E*_Eq_ and *E*_Inst_ and partition maps of CA4 + and gadoteridol were constructed to assess spatial variance and visualize the results (Fig. 3). For *E*_Eq_ and *E*_Inst_, the areas between the measured locations were solved using linear interpolation. The partition maps for Fig. 3c–f were created by summing up the pixel partition values in the image stack direction (axial).

### Statistical analysis

The normality of biomechanical data distribution was tested using the Kolmogorov–Smirnov test and the data was found non-normally distributed (*p* < 0.00001). For this reason, Spearman’s rank correlation coefficient was chosen to be used for the comparisons. A threshold of *p* < 0.05 was established as the level of statistical significance. The inspection was conducted separately for 20% depth increments (Fig. 6) and for bulk cartilage (i.e., full-thickness cartilage) partitions (Tables 1 and 2). DECT correlation analysis was done only for bulk cartilage due to the larger voxel size. The difference between the PCD-CT and DECT contrast agent diffusion time coefficients was studied using the Wilcoxon signed-rank test. The statistical analyses were performed with MATLAB (R2018b, MathWorks, Natick, MA, USA) using Statistics and Machine Learning Toolbox (version 11.4).

## Results

### PCD-CT and DECT validation

When using the LE and TE bins, the mean errors in estimated contrast agent concentration were 10.6% (*R*^2^ = 0.9918) and 2.4% (*R*^2^ = 0.9976) for CA4 + and gadoteridol, respectively, without the tibial phantom (Fig. 2a). When the tibial phantom was introduced into the scan, the mean errors for the CA4 + and gadoteridol were 9.6% (*R*^2^ = 0.9880) and 22.4% (*R*^2^ = 0.9600), respectively (Fig. 2b). For other bin combinations (without tibial phantom), the mean errors were higher: for LE and HE bins, the mean errors were 17.0% (*R*^*2*^ = 0.9850) and 16.2% (*R*^*2*^ = 0.9940) for CA4 + and gadoteridol, respectively, and, for HE and TE bins the mean errors were 22.3% (*R*^*2*^ = 0.9860) and 22.4% (*R*^*2*^ = 0.9930). For DECT, the mean errors in estimated concentration were 14.7% (*R*^2^ = 0.9945) and 6.3% (*R*^2^ = 0.9989) for CA4 + and gadoteridol, respectively (Fig. 2c).

### Biomechanical moduli and contrast agent partition heatmaps

The heatmaps of the biomechanical moduli and the contrast agent partitions are presented in Fig. 3. CA4 + partition exhibited qualitative correspondence with the *E*_Eq_. In both biomechanical moduli, areas with high variance of the values were observed. Qualitatively, similarities between CA4 + partition maps of PCD-CT and DECT were detected (Fig. 3c,e), but for gadoteridol, the partition maps between different scanners had high differences (Fig. 3d,f).

### Cartilage thickness, depthwise contrast agent profiles and diffusion

**Fig. 4:**
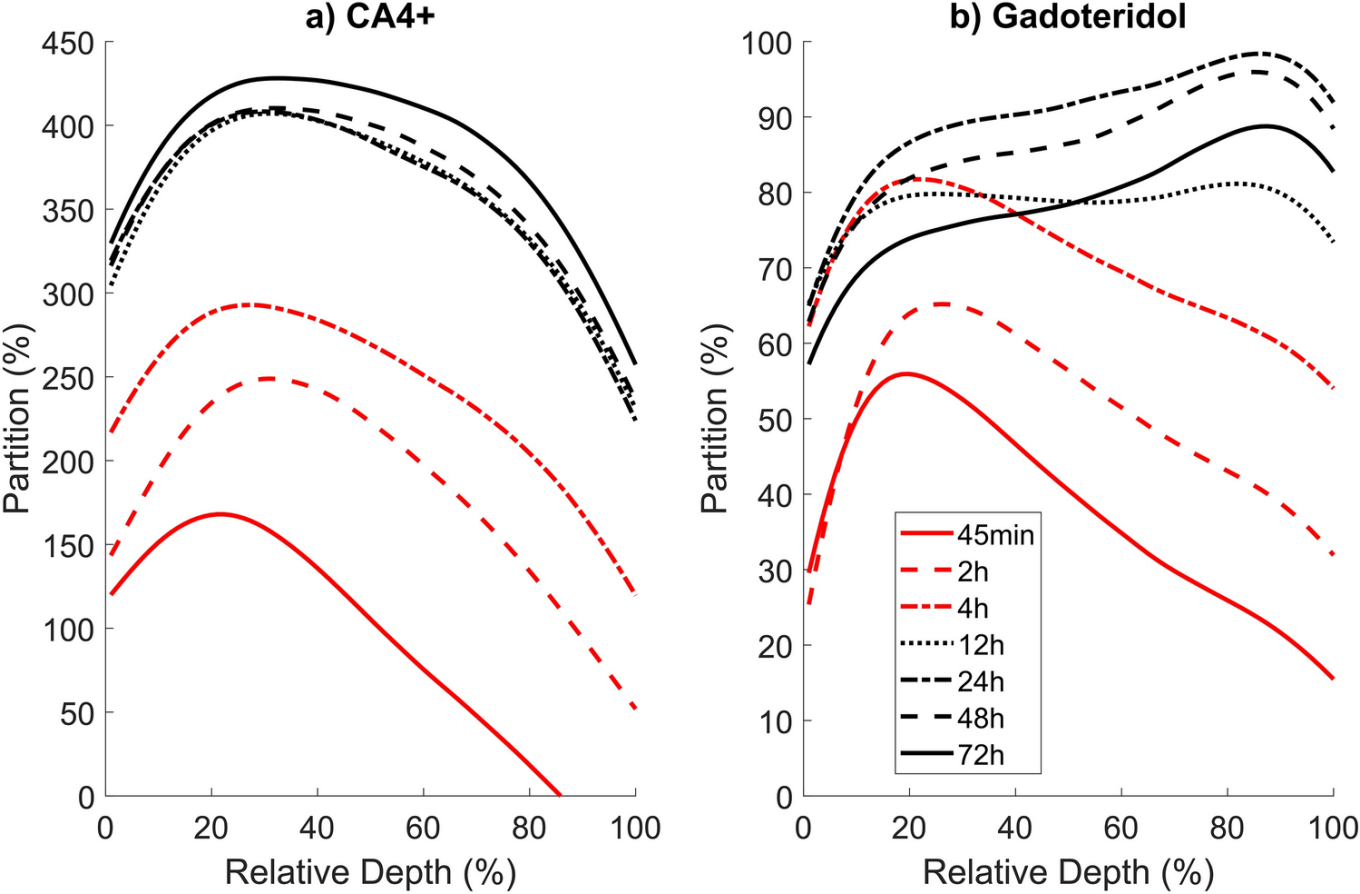
Depthwise contrast agent partition profiles for CA4 + (**a**) and gadoteridol (**b**) at different timepoints for photon-counting detector computed tomography (PCD-CT). Profiles are averaged from all the locations at each timepoint. The cartilage thickness is relative thickness calculated by linear interpolation and the cartilage surface is at 0% and the cartilage-bone interface is at 100%.

**Fig. 5:**
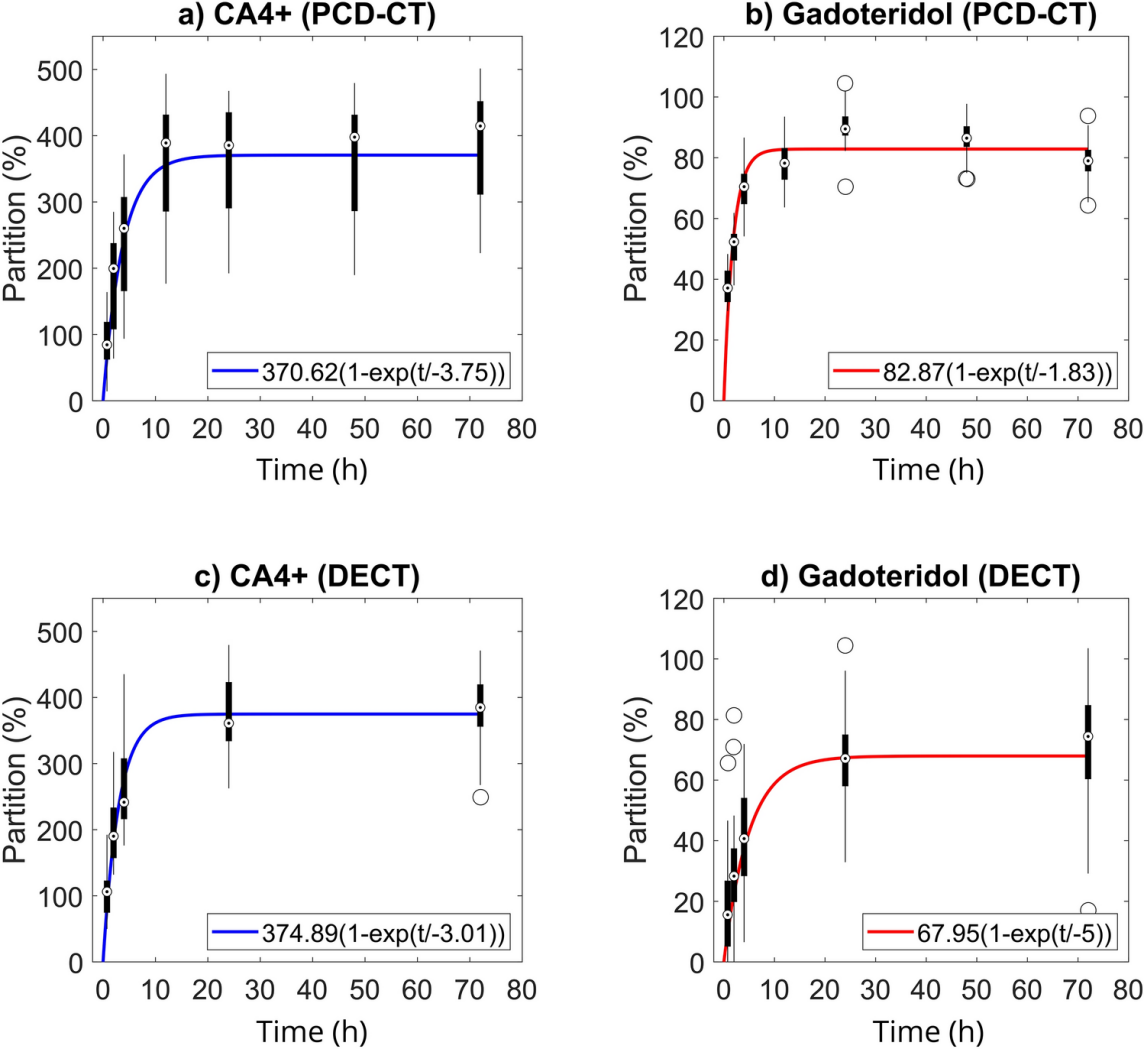
Experimental photon-counting detector computed tomography (PCD-CT, **a** and **b**) and clinical dual-energy computed tomography (DECT, **c** and **d**) contrast agent partitions of the bulk cartilage at different timepoints. Boxplots show the median with a dot, the 75th percentile with a thick black line, the interquartile range with a thin black line, and the outliers with circles. The diffusion data points for each measurement location were fitted separately. The obtained equation constants were averaged and plotted here. The 12- and 48-h timepoints were excluded from the DECT data. With PCD-CT, the equilibrium partitions were 371% and 83% for CA4 + and gadoteridol, respectively, and similarly, for DECT the values were 375% and 68%, respectively. Correspondingly, for PCD-CT CA4 + and gadoteridol diffusion time constants (the point where 63.2% of the equilibrium partition is reached) were 3.8 h and 1.8 h, respectively, and 3.0 h and 5.0 h for the DECT.

The average articular cartilage thickness at measured locations was 1.70 mm with a standard deviation of 0.36 mm. In the PCD-CT dataset, the CA4 + uptake increased in all cartilage depths up to the 72-h timepoint; the CA4 + partition was highest at the middle zone and lowest in the deep cartilage (Fig. 4). Gadoteridol profiles exhibited a similar shape up to a 4-h timepoint, but after that, the highest partition was reached in deep cartilage. The PCD-CT bulk partitions at 72-h timepoint were 386.48 ± 87.03% (mean ± standard deviation) and 78.76 ± 6.83% for CA4 + and gadoteridol, respectively. For DECT, the bulk partitions were 379.29 ± 52.91% and 70.22 ± 19.88%. The contrast agent partitions over time in bulk cartilage, determined with PCD-CT, are presented in Fig. 5a,b.

The CA4 + diffusion time constant *τ*^CA4+^ was 3.0 h (95% confidence interval (CI) [2.6, 3.4]) and 3.8 h (95% CI [3.2, 4.3]) for DECT and PCD-CT, respectively (Fig. 5a,c). For the gadoteridol, the diffusion time coefficient, *τ*^Gd^, measured with DECT was significantly higher (*p* < 0.0001) than that determined with the PCD-CT at 5.0 h (95% CI [3.5, 6,5]) and 1.8 h (95% CI [1.6, 2.0]), respectively (Fig. 5b,d).

### Correlation between biomechanical properties and contrast agent partitions estimated using PCD-CT

**Fig. 6:**
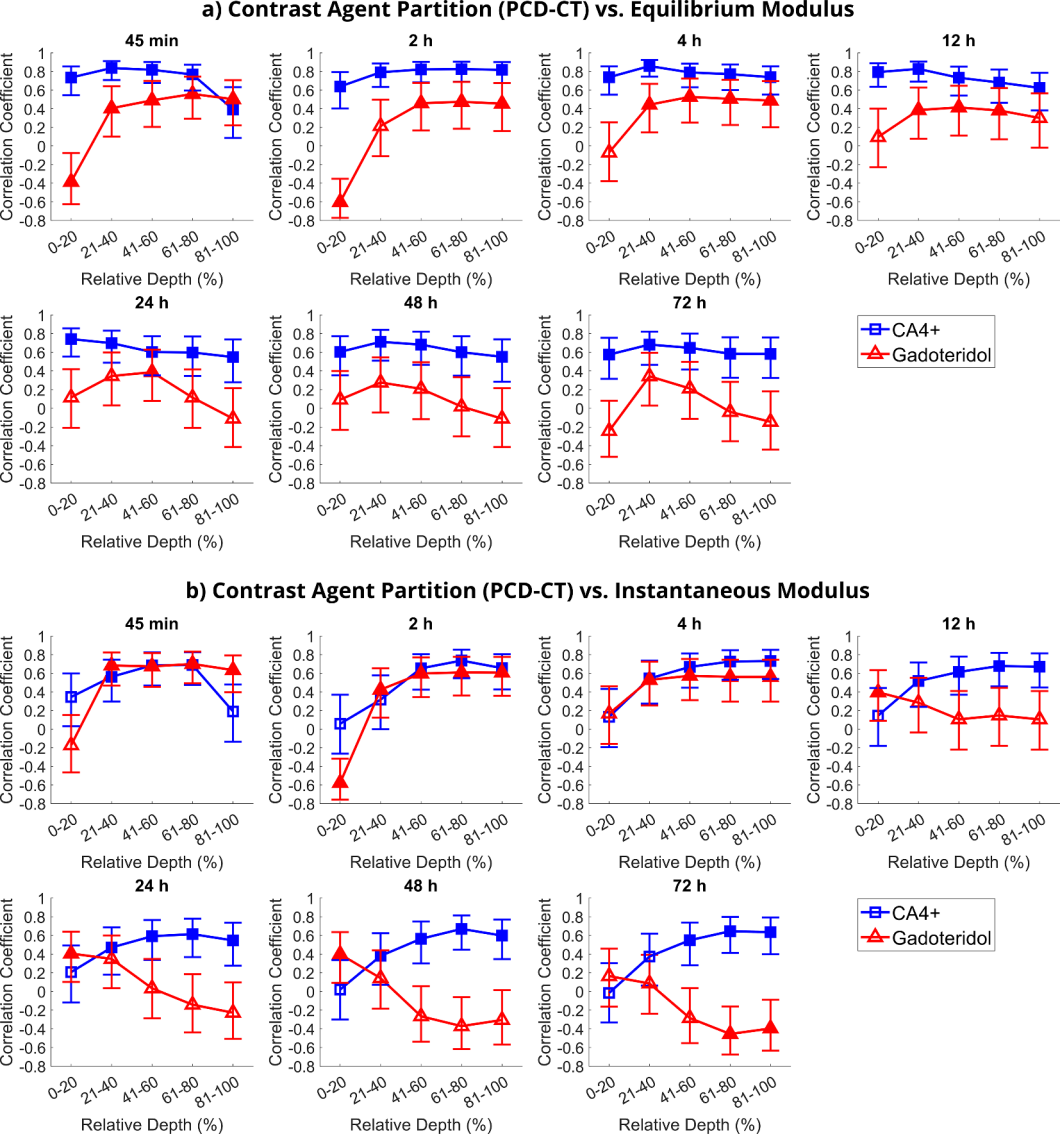
Depthwise Spearman’s correlation coefficients between equilibrium and instantaneous moduli and contrast agent partitions determined with photon-counting detector computed tomography (CT) in 20% intervals of cartilage thickness at different timepoints. For clinical dual-energy CT, the depthwise analysis was not possible due to large voxel size. A statistically significant correlation (*p* < 0.05) is marked with a filled marker. 95% confidence intervals are marked with vertical bars. The cartilage surface is at 0% and the cartilage-bone interface is at 100%.

Significant correlations between the CA4 + partition and *E*_Eq_ and *E*_Inst_ were found (Tables 1a and 2a). A strong positive correlation was observed between ${P}_{\text{max}}^{\text{CA}4+}$ and *E*_Eq_ in bulk cartilage (*ρ* = 0.68, 95% CI [0.46, 0.82], *p* < 0.0001) at a 72-h timepoint (Table 1a). Furthermore, the correlation between the CA4 + partition and *E*_Eq_ was moderate to strong at each timepoint and at each depth (Fig. 6a and Table 2a). In addition, *E*_Inst_ showed a significant correlation with CA4 + partition at all timepoints in bulk cartilage (Table 2a), but in depthwise analysis, the significant relationship was found mainly in mid to deep zones (Fig. 6b). There was a strong negative correlation between *τ*^CA4+^ and* E*_Eq_ in bulk cartilage (*ρ* = −0.85, 95% CI [0.92, −0.72], *p* < 0.0001), and similarly a moderate positive correlation between *τ*^CA4+^ and *E*_Inst_ and *β* (*ρ* = −0.56, 95% CI [−0.75, −0.30], *p* = 0.0019 and *ρ* = 0.57, 95% CI [0.30, 0.75], *p* = 0.0017, respectively). The *τ*^Gd^ and *E*_Inst_ had a strong negative correlation (*ρ* = −0.71, 95% CI [−0.84, −0.50], *p* < 0.0001) in bulk cartilage.

**Table 1:** Spearman’s correlation coefficients between the biomechanical data and the bulk contrast agent diffusion results of experimental photon-counting detector computed tomography (PCD-CT, **a**) and clinical dual-energy computed tomography (DECT, **b**). 95% confidence intervals are given in brackets.

	${{\varvec{P}}}_{\mathbf{m}\mathbf{a}\mathbf{x}}^{\mathbf{C}\mathbf{A}4+}$	${{\varvec{\tau}}}^{\mathbf{C}\mathbf{A}4+}$	${{\varvec{P}}}_{\mathbf{m}\mathbf{a}\mathbf{x}}^{\mathbf{G}\mathbf{d}}$	${{\varvec{\tau}}}^{\mathbf{G}\mathbf{d}}$
**(a) PCD-CT**				
***E***_**Eq**_	0.68*** [0.46, 0.82]	−0.85*** **[−0.92, −0.72]**	0.15 [**−**0.18, 0.44]	**−**0.35 [**−**0.60, **−**0.04]
***E***_**Inst**_	0.54** **[0.27, 0.73]**	−0.56** **[−0.75, −0.30]**	**−**0.06 [**−**0.37, 0.26]	−0.71*** **[−0.84, −0.50]**
***α***	**−**0.30 [**−**0.56, 0.02]	0.35 [0.04, 0.60]	**−**0.09 [**−**0.40, 0.23]	**−**0.36 [**−**0.61, **−**0.05]
***β***	−0.48** **[−0.70, −0.20]**	0.57** **[0.30, 0.75]**	**−**0.23 [**−**0.51, 0.10]	**−**0.14 [**−**0.43, 0.19]
**(b) DECT**				
***E***_**Eq**_	0.36 [0.05, 0.61]	−0.51** **[−0.71, −0.23]**	**−**0.26 [**−**0.54, 0.06]	0.01 [**−**0.31, 0.32]
***E***_**Inst**_	**−**0.24 [**−**0.52, 0.09]	−0.75*** **[−0.86, −0.57]**	**−**0.23 [**−**0.51, 0.10]	**−**0.31 [**−**0.57, 0.01]
***α***	−0.54** **[−0.73, −0.27]**	**−**0.19 [**−**0.48, 0.14]	**−**0.06 [**−**0.37, 0.26]	**−**0.27 [**−**0.54, 0.05]
***β***	−0.63*** **[−0.79, −0.40]**	0.06 [**−**0.26, 0.37]	0.10 [**−**0.22, 0.41]	**−**0.22 [**−**0.50, 0.11]

Statistically significant values are in bold.

Statistical significance: *p < 0.05; **p < 0.01; ***p < 0.001.

*P*_*max*_ Maximum contrast agent partition, *τ* Contrast agent diffusion time constant, *E*_*Eq*_ Equilibrium modulus, *E*_*Inst*_ Instantaneous modulus, *α* Relaxation time constant, driven by porosity, *β* Relaxation time constant, driven by solid content.

**Table 2:** Spearman’s correlation coefficients between biomechanical moduli and contrast agent bulk partitions of experimental photon-counting detector computed tomography (PCD-CT, a) and clinical dual-energy computed tomography (DECT, b), at different timepoints. Spearman’s correlation coefficients between PCD-CT and DECT contrast agent partitions at different timepoints are presented in sub-table (c). The 12- and 48-h timepoints were excluded from the DECT data due to failure in data acquisition. 95% confidence intervals are given in brackets.

a) PCD-CT				
Timepoint	*E*_Eq_		*E*_Inst_	
	CA4 +	Gd	CA4 +	Gd
**45 min**	0.81*** **[0.67, 0.90]**	0.43* **[0.13, 0.66]**	0.63*** **[0.39, 0.79]**	0.68*** **[0.47, 0.82]**
**2 h**	0.83*** **[0.70, 0.91]**	0.25 [**−**0.07, 0.53]	0.52** **[0.25, 0.72]**	0.42* **[0.12, 0.65]**
**4 h**	0.81*** **[0.67, 0.90]**	0.48* **[0.19, 0.69]**	0.64*** **[0.40, 0.79] **	0.52** **[0.25, 0.72]**
**12 h**	0.77*** **[0.59, 0.87]**	0.41* **[0.10, 0.64]**	0.57** **[0.31, 0.75]**	0.25 [**−**0.07, 0.53]
**24 h**	0.69*** **[0.48, 0.83]**	0.14 [**−**0.18, 0.44]	0.56** **[0.29, 0.74]**	0.11 [**−**0.22, 0.41]
**48 h**	0.69*** **[0.47, 0.82]**	0.04 [**−**0.28, 0.35]	0.52** **[0.24, 0.72]**	**−**0.15 [**−**0.44, 0.18]
**72 h**	0.64*** **[0.40, 0.79]**	**−**0.03 [**−**0.34, 0.29]	0.51** **[0.23, 0.71]**	**−**0.27 [**−**0.54, 0.06]

**b) DECT**				
**Timepoint**	***E***_**Eq**_		***E***_**Inst**_	
	**CA4 + **	**Gd**	**CA4 + **	**Gd**
**45 min**	0.48** **[0.20, 0.70]**	0.02 [**−**0.30, 0.33]	0.32 [**−**0.01, 0.58]	0.33 [0.02, 0.59]
**2 h**	0.68*** **[0.47, 0.82]**	**−**0.13 [**−**0.43, 0.20]	0.53** **[0.26, 0.73]**	**−**0.01 [**−**0.32, 0.32]
**4 h**	0.68*** **[0.46, 0.82]**	**−**0.06 [**−**0.37, 0.26]	0.46* **[0.17, 0.68]**	0.12 [**−**0.21, 0.42]
**12 h**	–	–	–	–
**24 h**	0.50** **[0.21, 0.70]**	**−**0.07 [**−**0.38, 0.25]	**−**0.15 [**−**0.44, 0.18]	0.10 [**−**0.23, 0.40]
**48 h**	–	–	–	–
**72 h**	0.20 [**−**0.12, 0.49]	**−**0.15 [**−**0.44, 0.18]	**−**0.36 [**−**0.61, **−**0.04]	**− **0.13 [**−**0.43, 0.19]

**c) PCD-CT vs. DECT**				
Timepoint	Partition			
	CA4 +	Gd		
**45 min**	0.52** **[0.25, 0.72]**	0.24 [**−**0.08, 0.52]		
**2 h**	0.60*** **[0.34, 0.77]**	**−**0.35 [**−**0.60, **−**0.04]		
**4 h**	0.62*** **[0.37, 0.78]**	**−**0.26 [**−**0.54, 0.06]		
**12 h**	–	–		
**24 h**	0.21 [**−**0.12, 0.49]	**−**0.07 [**−**0.38, 0.26]		
**48 h**	–	–		
**72 h**	**−**0.06 [**−**0.37, 0.26]	**−**0.18 [**−**0.47, 0.15]		

Statistically significant values are in bold.

Statistical significance: *p < 0.05; **p < 0.01; ***p < 0.001.

*E*_*Eq*_ Equilibrium modulus, *E*_Inst_ Instantaneous modulus.

### Correlation between biomechanical properties and contrast agent partitions estimated using DECT

${P}_{\text{max}}^{\text{CA}4+}$ Had a significant correlation with *α* and *β* (*ρ* = −0.54, 95% CI [−0.73, −0.27], *p* = 0.0030 and *ρ* = −0.63, 95% CI [−0.79, −0.40], *p* = 0.0003, respectively). *τ*^CA4+^ had a significant correlation with *E*_Eq_ and *E*_Inst_ (*ρ* = −0.51, 95% CI [−0.71, −0.23], *p* = 0.0059 and *ρ* = −0.75, 95% CI [−0.86, −0.57], *p* < 0.0001, respectively) (Table 1b). Early timepoints (≤ 4 h) in the DECT dataset showed the same link between biomechanical moduli and CA4 + bulk partition as in the PCD-CT dataset (Table 2b). Between *E*_Eq_ and CA4 + partition, the correlation coefficient ranged between *ρ* = 0.48 and *ρ* = 0.68, being strongest at the 2- and 4-h timepoints, but no significant relationship was found any more at the 72-h timepoint. For the *E*_Inst_, a significant correlation was detected only at 2- and 4-h timepoints, with a correlation coefficient of 0.53 (95% CI [0.26, 0.73], *p* = 0.0035) and 0.46 (95% CI [0.17, 0.68], *p* = 0.0141), respectively.

### Correlation between PCD-CT and DECT contrast agent partitions

The CA4 + partitions in bulk cartilage determined with PCD-CT and DECT correlated at 45-min (*ρ* = 0.52, 95% CI [0.25, 0.72], *p* = 0.0042), at 2-h (*ρ* = 0.60, 95% CI [0.34, 0.77], *p* = 0.0008), and at 4-h timepoint (*ρ* = 0.62, 95% CI [0.37, 0.78], *p* = 0.0005) (Table 2c). No significant relationship was found between gadoteridol partitions with different CT techniques.

## Discussion

Based on the results of our proof-of-concept study, PCD-CT was found to assess the biomechanical properties of articular cartilage via a dual-contrast agent method with greater sensitivity and accuracy than a clinical DECT scanner, supporting our initial hypothesis. According to the validation results without tibial phantom, PCD-CT provided more accurate and precise material decomposition compared to DECT (Fig. 2). After the introduction of the tibial phantom, PCD-CT’s determination was only minimally affected. The PCD-CT scanner also enabled higher spatial resolution. Based on the present results (Figs. 2, 3 and 5), DECT was not as sensitive and reliable as PCD-CT in assessing contrast agent partitions, especially gadoteridol partitions.

As anticipated, the maximum partition of CA4+, ${P}_{\text{max}}^{\text{CA}4+}$, showed a positive correlation with the biomechanical moduli (Table 1a), which is consistent with the fact that the depthwise CA4 + partition is known to mirror the cartilage’s physiological PG distribution^12,16,36,38–40^ due to the cationic CA4 + molecule being electrostatically attracted to the negative fixed charge density formed by PGs^15,18,26,45–47^. PGs modulate the cartilage equilibrium Young’s modulus through osmotic pressure^48,49^, and thus, the number of PGs is directly proportional to the stiffness. CA4 + time constant *τ*^CA4+^, negatively correlated with *E*_Eq_ in bulk cartilage, which was expected since the* τ*^CA4+^ represents the time CA4 + reaches equilibrium (Table 1a). Based on the result, higher PG content in cartilage (represented by *E*_Eq_) can increase CA4 + flux allowing equilibrium to be reached faster. Similarly, *τ*^Gd^ negatively correlated with *E*_Inst_, suggesting that a more organized collagen network (contributing to *E*_Inst_) facilitates a more unobstructed flow of the neutral molecules. CA4 + (${P}_{\text{max}}^{\text{CA}4+}$ and *τ*^CA4+^) was also able to reflect the relaxation time constant *β*, which reflects the strain-dependency of the extracellular tissue permeability. *β* is low (i.e., mechanical equilibrium under prolonged loading is reached faster) in denser samples that have more PGs, i.e., high ${P}_{\text{max}}^{\text{CA}4+}$, and small *τ*^CA4+^. The observed correlations between the CA4 + and biomechanical moduli agreed with the results from earlier studies done with PCD-CT^26^, micro-CT^18,35,46,50,51^, and synchrotron micro-CT^45^. The correlation of CA4 + partition with *E*_Eq_ stayed strong and positive at all timepoints and in all cartilage zones (Fig. 6a). *E*_Inst_ correlated with CA4 + partition mainly in mid to deep zones (Fig. 6b), which have higher collagen content.

When looking at the depth-dependent results, gadoteridol partition negatively correlated with both biomechanical moduli in the superficial zone^18,26^, albeit only at 45-min and 2-h timepoints with *E*_Eq_ and 2-h timepoint with *E*_Inst_ (Fig. 6). These early negative correlations are due to stiffer samples having higher collagen content and lower porosity in the superficial zone^52^. We expect that CA4 + (contributing to beam hardening) might cause an error in the partition estimation of gadoteridol after two hours, especially in deep cartilage which attracts more CA4 + . Unexpectedly, gadoteridol partition was significant and positively correlated with biomechanical moduli in the middle zone between the 45-min and 24-h timepoints (Fig. 6). Even though average diffusion was seen to stabilize by this time, concentrations continue to change in deeper cartilage^52^. At the 24-h timepoint, the fitted bulk CA4 + partition for PCD-CT data reached 99.83% of the ${P}_{\text{max}}^{\text{CA}4+}$ (Fig. 5a). At the 12-h timepoint, the fitted bulk gadoteridol partition had reached 99.86% of the ${P}_{\text{max}}^{\text{Gd}}$, after which a decrease was observed in measured data (Figs. 4b, 5b). This decrease in the measured partition data might be caused by errors in the material decomposition or the increasing CA4 + partition (Fig. 4a), which likely distorted the gadoteridol’s depthwise profiles at timepoints after 12 h (Fig. 4b). Similarly, the fitted bulk CA4 + partition for the DECT data reached 99.97% of the ${P}_{\text{max}}^{\text{CA}4+}$ (Fig. 5c) at the 24-h timepoint, but the fitted bulk gadoteridol partition had reached only 90.93% of the ${P}_{\text{max}}^{\text{Gd}}$ at the 12-h timepoint (Fig. 5d).

Between DECT and PCD-CT datasets, significant correlations were found only before the 4-h timepoint for the CA4 + partition. Still, the analysis of the DECT dataset produced comparable correlation results with PCD-CT, such as a high positive correlation between the CA4 + partition and *E*_Eq_. However, for DECT, the significance was lost after 24 h. Between the CA4 + partition and *E*_Inst_, the correlation was significant only at the 2- and 4-h timepoints. Based on the *ex vivo* results, the optimal imaging time could be between two and four hours since the correlations were at the strongest around those timepoints in both CT systems. However, *in vivo* studies are needed to confirm this since this study was done *ex vivo* and did not consider the effect of synovial fluid and metabolism.

The gadoteridol partition of DECT did not significantly correlate with any of the reference parameters. A similar lack of correlation has been previously reported in intact cadaveric knee joints with clinical DECT scanner^53^. The authors suspect that the most likely suspect for this was beam hardening that caused error into the material decomposition. The inclusion of a tibial phantom in the validation images for PCD-CT also demonstrated that higher attenuation and a harder beam led to underestimated contrast agent concentrations (Fig. 2a,b) and significantly increased errors in gadoteridol estimation in PCD-CT measurements (mean error of 22.4% and 2.4% for gadoteridol, with and without the tibial phantom, respectively, Fig. 2a,b). This indicates that the gadoteridol is sensitive to beam hardening caused by the tibial phantom, thus, it can be challenging to use this contrast agent combination in a clinical environment. This observation is valuable and could explain the weak or non-significant correlations between gadoteridol and reference data for both imaging modalities, emphasizing the impact of beam hardening on the sensitivity of the calibration-based material decomposition method, which should be taken into account when imaging larger samples or intact knee joints. This is especially true for the DECT (Fig. 3f) and this was also reported in a study by Orava et al.^53^. Similarities in the CA4 + distribution and partition were observed in the heatmaps (Fig. 3c,d) between PCD-CT and DECT, while the gadoteridol heatmaps (Fig. 3e,f) did not exhibit such similarities. Moreover, the DECT heatmap has overestimated partition values as non-ionic gadoteridol cannot reach partitions over 100% as the diffusion is based on osmosis. Therefore, the gadoteridol should follow the water content of articular cartilage which is typically between 65 and 80%^54,55^. Additionally, the imaging setups are different and the main differences are the detector technology (one X-ray spectrum with PCD-CT vs. two X-ray spectra with DECT resulting in different energy bins and, therefore, different material decomposition) and voxel size (68.4 × 68.4 × 68.4 µm^3^ with PCD-CT vs. 0.32 × 0.32 × 0.60 mm^3^ with DECT causing a stronger partial volume effect in DECT data). The effect of the detector technology is also visible in the calibration data (Fig. 1), where the calibration curves of CA4 + share similarities between PCD-CT and DECT, but the calibration curve of gadoteridol has very distinctive differences. This is due to differences between detector technologies. In PCD-CT data, gadoteridol has slightly higher attenuation at the high energy bin, as in DECT data, the low energy attenuates always more than high energy with both contrast agents. However, this is not a problem but an advantage because our calibration-based material decomposition method is more robust when the attenuation of one contrast agent is higher at one energy and lower at the other energy compared to another contrast agent. Nonetheless, the beam hardening constraints in both modalities could be addressed through the implementation of even more robust methods, such as deep-learning-based material decomposition^56^ or improved artifact correction^57^, or by exploring different contrast agent combinations.

In this proof-of-concept study, extensive and challenging measurements were done for one *ex vivo* bovine medial tibial plateau. This is a limitation and it was addressed by maximizing the number of biomechanical measurement locations on the sample. The variance observed in the biomechanical moduli (Fig. 3a,b) supports the suggestion that the chosen locations have sufficient differences in the structure and function of articular cartilage to enable the analysis. The current findings, derived from a healthy sample, do not offer insights into the ability of the methodology to detect post-traumatic OA. These results in healthy cartilage lay a pivotal groundwork for future investigations. However, it is worth noting that the observed variation in biomechanical properties aligns with changes typically observed in the early stages of the disease^58,59^ and that the current PCD-CT correlation results are consistent with previous studies using the same contrast agent combination and osteoarthritic samples^18,26,60^. Even though the contrast agent bath temperature was set to 10 °C, which slows down the diffusion (based on thermodynamics), it had no significant effect on the interpretation of the results since the sample did not degenerate significantly due to the temperature and inhibitors in the bath, and because the diffusion equilibrium was achieved.

PCDs suffer from common and unavoidable limitations, such as cross talk, charge sharing, pulse pile-up, and *K*-escape^1^. In the current study, these limitations were addressed by using a software-based solutions to correct charge sharing, keeping photon flux low, and lengthening the imaging time to reduce pulse pile-up. Dead pixels are common in PCDs due to difficulties in manufacturing conversion media, and thus, ring artifacts were evident in the reconstructed images (Fig. 3c,d). However, STC calibration reduced their amount. Some ring artifacts were still present in the final summed image stacks due to material decomposition but those had minimal effect on the results.

## Conclusions

A dual-contrast agent method using an experimental two-bin PCD-CT setup differentiates the concentrations of two contrast agents concentrations within the *ex vivo* bovine medial tibial plateau articular cartilage. We investigated this scenario to account for increased beam hardening and scattering caused by the bone surrounding the cartilage, as previous studies focused only on smaller osteochondral plugs. Based on the results, the dual-contrast agent approach functions well with spectral PCD-CT. However, it is crucial to acknowledge that the presence of surrounding tissue, leading to beam hardening and scattering, significantly influences the accuracy of material decomposition for gadoteridol. To address this limitation, exploration of potential improvements could involve investigating alternative attenuating elements to substitute gadolinium or optimizing the PCD bins. For instance, novel nanoparticles present a promising avenue because of their unique property—the capability to choose the core element metal^61^. This property allows for tailored compositions that may offer enhanced performance in mitigating beam hardening and scattering effects.

In summary, PCD-CT successfully assesses the biomechanical properties of articular cartilage via the dual-contrast agent diffusion with strong and significant correlations between the CA4 + contrast agent and *E*_Eq_. In addition, PCD-CT provides visual maps to quantify different regions of cartilage where early osteoarthritic changes are present. Spectral PCD-CT combined with the dual-contrast agent method outperforms DECT in terms of accuracy and sensitivity. However, further research is necessary to fully explore the potential of the PCD-CT dual-contrast agent method, particularly in applications such as diagnosing post-traumatic OA. Nevertheless, PCD-CT offers substantial potential, and its continued development may lead to a novel minimally invasive CT imaging technique for assessing various medical conditions. 

## Data Availability

All data needed to evaluate the conclusions in the paper are present in the paper. Additional data related to this paper may be requested from the authors.
